# Validation of phenology models for *Halyomorpha halys* (Hemiptera: Pentatomidae) using field data from climatically different ecoregions

**DOI:** 10.1093/ee/nvaf097

**Published:** 2025-10-08

**Authors:** Emily C Ogburn, Stephen C Schoof, Dominic D Reisig, George G Kennedy, James F Walgenbach

**Affiliations:** Department of Entomology and Plant Pathology, NC State University, Mountain Horticultural Crops Research and Extension Center, Mills River, NC, USA; Department of Entomology and Plant Pathology, NC State University, Mountain Horticultural Crops Research and Extension Center, Mills River, NC, USA; Department of Entomology and Plant Pathology, NC State University, Vernon G. James Research and Extension, Center, Plymouth, NC, USA; Department of Entomology and Plant Pathology, NC State University, Raleigh, NC 27695, USA; Department of Entomology and Plant Pathology, NC State University, Mountain Horticultural Crops Research and Extension Center, Mills River, NC, USA

**Keywords:** brown marmorated stink bug, model validation, degree-day

## Abstract

*Halyomorpha halys* (Stål), an invasive species of Asian origin, has become a key pest of tree fruit in areas of the Eastern and Pacific Northwest United States. This study used a 5-yr dataset of pheromone trap captures from 4 ecoregions (Mountains, Piedmont, Southeastern Plains, and Atlantic Coastal Plain) of North Carolina to validate temperature-driven phenology models previously reported for oviposition by overwintering adults and eclosion of F1 adults using semi-field cage studies. Cumulative proportion of pheromone trap captures of F1 adults and nymphs over cumulative degree days was compared to predictions based on the previously reported models’ quadratic equation curves for adult eclosion (*y *= −0.0000015*x*^2^ + 0.004736*x* − 2.664) and oviposition (*y *= 0.0000032*x*^2^ − 0.010853*x* + 9.050). The oviposition model was validated using projected oviposition curves to predict nymphal populations over time by using life stage-specific development and mortality rates. Analysis of coefficients of determination (*R*^2^) for all regressions showed that F1 adult model predictions varied by region and year. Mean *R*^2^ values in the Mountain, Piedmont, and Southeastern Plains ecoregions for F1 adults were 0.88, 0.93, and 0.93, respectively. Nymphal regressions also varied by ecoregion, with mean *R*^2^ values of 0.95, 0.86, and 0.88 in the Mountains, Piedmont, and Southeastern Plains, respectively. Differences among regions were mostly associated with lower *R*^2^ values at sites with low population densities. Results are discussed in relation to the value of these models in studying the ecology of invasive species and in informing pest management decisions.

## Introduction

Phenology models are valuable tools for predicting voltinism and generational development of insect species (Rebaudo and Rabhi 2018). The temporal predictions of population age structure, heightened pest pressure, and/or damage risk that they provide are key to making informed decisions for targeted pest management plans (Welch et al. 1978, Kemp and Dennis 1991, Crimmins et al. 2020, Cassidy et al. 2022). Phenology model-based timing of pesticide applications has led to improved efficiency of pesticide use for a range of pest populations (Way and van Emden 2000, Jones et al. 2010).

Temperature is a key factor affecting the development of poikilothermic organisms that can lead to differences in seasonal phenology among areas with differing local environmental conditions and/or climate (Bale et al. 2002, Powell and Logan 2005, Rebaudo and Rabhi 2018). Temperature-based phenology models can be utilized to understand and predict insect population dynamics in invaded ranges and in areas with shifting environmental conditions due to climate change (Jarvis and Baker 2001, Skendžić et al. 2021, Cassidy et al. 2022). Phenology models are also useful in developing geographic range suitability models that predict areas vulnerable to expanding or shifting distributions of invasive pests (Fand et al. 2014, Bentz et al. 2019, Barker et al. 2020, Takeuchi et al. 2023).

Native to East Asia, the brown marmorated stink bug (*Halyomorpha halys* Stål, BMSB) has become a widespread invasive agricultural and nuisance pest for which phenology models have proven useful for studying population dynamics in its invaded range (Rice et al. 2014, Haye et al. 2015, Nielsen et al. 2016). Overwintering as adults in facultative diapause, BMSB shelters in protected areas including human-made structures (Lee et al. 2014a), and voltinism varies across its invaded range (Haye et al. 2015, Ingels and Daane 2018, Ogburn et al. 2023). Using BMSB from Pennsylvania, the founder population for eastern North American (Xu et al. 2014, Valentin et al. 2017), Nielsen et al. (2008) estimated lower and upper threshold temperatures as 14.17 and 35.76 °C, respectively, and estimated stage-specific developmental and mortality rates for the 5 immature life stages after egg hatch. Subsequently, Nielsen et al. (2016) used an agent-based stochastic model to simulate life-stage specific phenology and population density at 8 locations in the United States, using a photoperiod of 13.5 h to trigger both induction and termination of diapause. The model predicted a high degree of overlap among life stages and generations and bivoltine populations at all locations, with F2 generation adults comprising the majority of the overwintering population (Nielsen et al. 2016). Overlapping generations of BMSB make field-based comparisons and validations of phenology across regions difficult; therefore, Nielsen et al. (2017) added reproductive physiology to modeling efforts by examining seasonality of female reproductive stages at locations in 5 states. They found that following diapause termination in the spring, there was a delay before females became reproductively mature, and that a 12.7 h photoperiod in the spring, when 10% of females became reproductively mature, was the best fit for a biofix in temperate regions (Nielsen et al. 2017). Subsequently, McDougall et al. (2021) proposed a critical photoperiod of 13.5 h, when 50% of females reached reproductive maturity, as an alternative biofix for BMSB populations in the eastern United States.

An empirical phenology model based on BMSB development under semi-field conditions in two North Carolina ecoregions having the same photoperiod but different temperature profiles showed that BMSB was univoltine in the Mountain region and bivoltine in the warmer Southeastern Plains region (Ogburn et al. 2023). These models described cumulative oviposition and eclosion of adult generations, separately, over cumulative degree-days from the 2 proposed biofixes –12.7 h (Nielsen et al. 2017) and 13.5 h photoperiods (McDougall et al. 2021). Quadratic equations best fit both relationships (Ogburn et al. 2023), and while both biofixes resulted in significant models, variation between ecoregions was less with the 12.7 h photoperiod biofix.

**Fig. 1.**
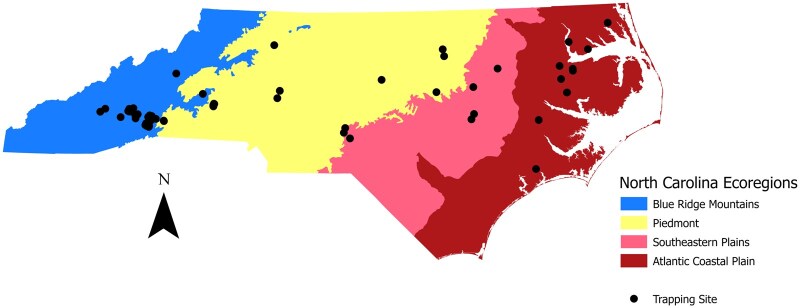
Map of *H. halys* trapping locations across ecoregions of North Carolina. Map generated using NC level III shapefile (US Environmental Protection Agency 2012).

The objective of this study was to build on phenology modeling efforts of BMSB in its invaded range and validate the BMSB phenology models developed for North Carolina populations (Ogburn et al. 2023). North Carolina is at the southern extent of the US range in which BMSB is a severe agricultural pest (stopBMSB.org). Large populations are well established in the western Blue Ridge Mountain and the Piedmont ecoregions, but low adult numbers and lack of nymphal presence in much of the Atlantic Coastal Plains ecoregion suggest populations have not established there (Bakken et al. 2015, Ogburn et al. 2023). Moving from the Mountains to Atlantic Coastal Plains ecoregions, elevation declines and temperatures increase (Fig. 1). Using 5 yr of BMSB pheromone trapping data collected at 87 NC field locations across the Blue Ridge Mountain (Mountains), Piedmont, Southeastern (SE) Plains, and Atlantic Coastal Plain (Coastal Plain) ecoregions, this study investigated the validity of the phenology models developed by Ogburn et al. (2023).

## Materials and Methods

### Trapping

Pheromone traps were deployed at locations in the Mountains, Piedmont, SE Plains, and Coastal Plain regions of North Carolina from 2017 to 2021 (Fig. 1). Both pyramid and sticky panel traps (Acebes-Doria et al. 2020) were used in 2017, but only sticky panel traps in subsequent years. Traps were baited with Dual Stink Bug Lures (Trece, Adair, Oklahoma) containing 5 mg of BMSB pheromone (Khrimian et al. 2014) and 50 mg of methyl (2E, 4E, 6Z)-decatrienoate (Weber et al. 2017). Two to 3 traps spaced ca. 50 m apart were established at each sampling location in April or May when adults emerged from overwintering sites. Traps were monitored through mid-October when dispersal to overwintering sites was complete. Traps were placed at the interface of agricultural crops (eg apple, corn, soybean, tomato) and adjacent wooded habitats and checked at ca. weekly intervals to record the number of BMSB adults and nymphs. Sticky cards were replaced as necessary to maintain a clean surface, and lures were replaced at 12-wk intervals (Acebes-Doria et al. 2020).

#### Model Parameters

Sampling locations in which cumulative trap captures averaged <25 adults or <40 nymphs during the predicted time of occurrence (see below) were excluded from the adult and nymphal model validation analyses, respectively. Comparatively, these were very low numbers, and captures at these sites were usually of individuals at seemingly random times during the year. We interpreted this as sites where BMSB was not locally established, due to the absence of suitable hosts or microclimates, or because the pest had not yet invaded the area. The models were intended to predict the phenology of established BMSB populations, and, therefore, regressions with low and sporadic trap captures did not yield meaningful information. Cumulative BMSB growing degree days were calculated starting from a biofix date of 4 April (12.7 h photoperiod in North Carolina) using the sine-wave method (Baskerville and Emin 1969) with upper and lower temperature thresholds of 14.17 and 35.76 °C, respectively (Nielsen et al. 2008). Temperature data were retrieved from the North Carolina State Climate Office (https://products.climate.ncsu.edu/) using the official weather station nearest to individual sites.

### Adult Eclosion Model Validation

#### F1 Adults

Adult pheromone trap capture data were used to validate the F1 adult eclosion model of Ogburn et al. (2023) that predicted cumulative proportion of eclosed F1 adults based on a quadratic function between 732 (predicted first emergence of F1 adults) and 1,356 (predicted first emergence of F2 adults) cumulative DD from a biofix date of 1 April. Pheromone trap captures for each location were converted to cumulative proportion captured for each sample date between 732 and 1,356 DD (1 April biofix). Trap data were limited to this interval, because pheromone trap captures included both overwintered and F1 adults, which could not be differentiated. Hence, based on Ogburn et al. (2023), it was logically assumed that only overwintered adults were captured before 732 DD, so these captures were disregarded. Model predictions for F1 adult eclosion were calculated by using the quadratic equation *y *= −0.0000015*x*^2^ + 0.004736*x* − 2.664, where *x* = degree days accumulated on the day when trap captures were recorded, and *y* = the model prediction for proportion of F1 adults eclosed by *x*.

General linear models (GLMs) in SAS (SAS Institute Inc. 2012. v.9.4) were used to examine the relationship between observed cumulative proportion F1 adults captured and the cumulative proportion predicted by the F1 adult eclosion model. The resultant *R*^2^ values from all locations were analyzed for significant differences among years and region using GLMs in SAS. Post hoc tests were conducted using Least-squares means with a Tukey–Kramer adjustment for multiple comparisons. Pyramid traps were only deployed in 2017; therefore, resultant *R*^2^ values only from 2017 sites in which both pyramid traps and sticky cards were deployed were analyzed using GLM for significant differences between trap types.

#### F2 Adults

Because later season BMSB pheromone trap captures potentially could include both F1 and F2 adults, which could not be differentiated, only adults captured after 1,356 DD (first predicted F2 adult eclosed, Ogburn et al. 2023) were considered to be F2 generation adults. Model predictions for F2 adult eclosion were calculated using the quadratic equation: *y *= 0.0000032*x*^2^ – 0.010853*x* + 9.050 (Ogburn et al. 2023), where *x* = degree days accumulated on the day on which the trap captures were recorded, and *y* = the model prediction for the proportion of F2 adults eclosed by *x.* The validation procedures outlined for the F1 adult eclosion model were used on cumulative F2 adult trap capture data (after 1,356 DD) to examine the cumulative proportion F2 adult capture versus F2 adult model prediction using GLMs. The *R*^2^ values produced were analyzed as dependent variables with year and trap capture as factors using GLMs. Only sticky trap captures were analyzed for F2 adults.

### Oviposition Model Validation

**Fig. 2.**
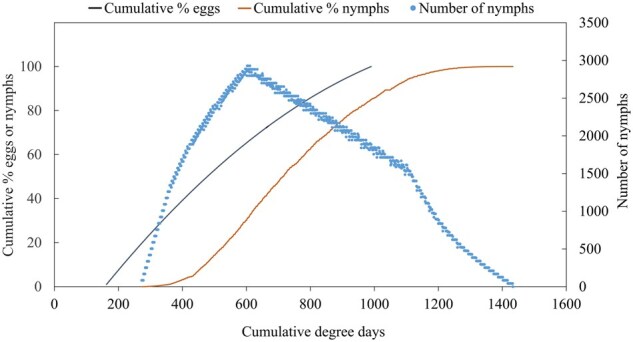
Predicted cumulative percentage of BMSB F1 eggs oviposited (Ogburn et al. 2023 model), and number and cumulative percentage of nymphs (instars 2 to 5) based on life-stage specific development and mortality rates (Nielsen et al. 2008) over time based on an F1 cohort of 10,000 eggs.

In the field, BMSB eggs are laid on the undersides of leaves of a diversity of host plants across complex landscapes, making it infeasible to collect sufficient BMSB oviposition data to validate the oviposition model. Hence, nymphal pheromone trap capture data were used to validate the oviposition model by extrapolating oviposition model predictions to predict nymphal population abundance over time and compare results with captures of nymphs in pheromone traps. To generate nymphal population curves, the F1 oviposition model (Ogburn et al. 2023) was used to generate an oviposition curve over time (DD) and egg and instar-specific development and mortality rates from Nielsen et al. (2008) were applied to estimate the abundance of instar-specific nymphs present over time. We assumed 10,000 eggs were oviposited between 163 and 990 DD (Ogburn et al. 2023) to begin the projection of numbers of nymphs present over time (Fig. 2). First instars do not disperse from eggs (Leskey et al. 2012, Lee et al. 2014b) and were not captured in pheromone traps, therefore only instars 2–5 were included in the cumulative number for the model prediction. The total number of nymphs was then expressed as cumulative proportion over time (DD) and compared to observational data of cumulative proportion nymphs captured in pheromone traps over time. To exclude potential F2 nymphs that may have been captured in traps, the model was terminated at 1,435 DD, which was the predicted timing of adult eclosion of the last F1 nymph.

General linear models (GLMs) were used to regress cumulative proportion of F1 nymphs captured in pheromone traps versus cumulative proportion F1 nymphal model predictions using SAS (SAS Institute Inc. 2012. v.9.4). Resultant *R*^2^ values produced by regressing these proportions for all locations were analyzed by region and year using GLMs in SAS. Post hoc tests were conducted using Least-squares means with a Tukey–Kramer adjustment for multiple comparisons. Resultant *R*^2^ values from 2017 sites in which both pyramid traps and sticky cards were deployed were analyzed for significant differences between trap types using GLMs.

## Results

### Trapping

**Fig. 3.**
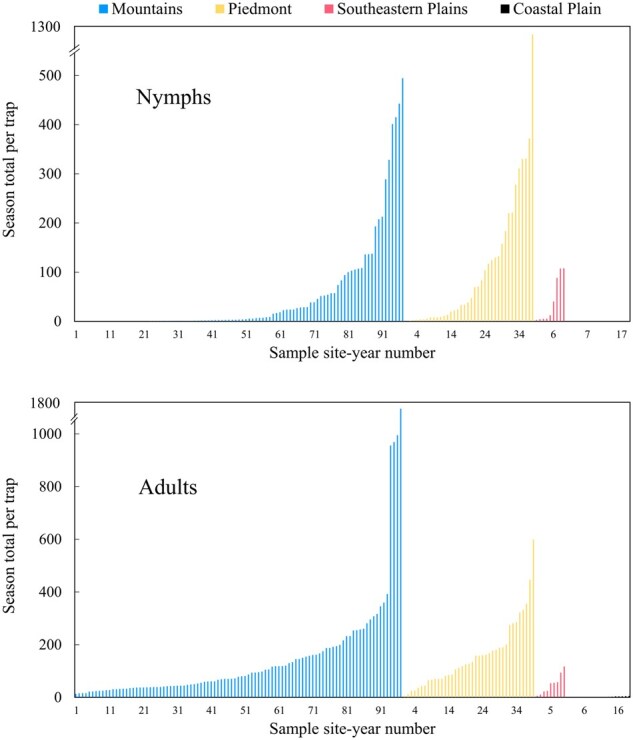
Season total nymphal and adult BMSB trap captures from different site-years in the Blue Ridge Mountains, Piedmont, Southeastern Plains, and Atlantic Coastal Plain of North Carolina, 2017 to 2021. Atlantic Coastal Plain had no nymphal trap capture and 0 to 4.5 adults per site-year.

BMSB captured in pheromone traps varied considerably among regions (Fig. 3). Across all sites per ecoregion, mean season total captures in the Mountains were 145.1 (±20.3) adults and 50.5 (±10.4) nymphs, in the Piedmont 156.9 (±20.3) adults and 107.9 (±22.0) nymphs, in the Southeastern Plains 48.1 (±12.6) adults and 41.1 (±15.5) nymphs, and in the Coastal Plain 1.2 (±0.4) adults and 0 nymphs.

### Adult Eclosion Model Validation

#### F1 Adults

The combined number of site-years with sticky trap captures adequate for F1 adult population model validation was 65, 26, 4, and 0 sites in the Mountains, Piedmont, Southeastern Plains, and Coastal Plain, respectively. Coastal Plain sites were excluded from model validation due to low F1 adult captures and no nymphal captures, showing there were no sites with established populations. The site with the highest season total capture of all adults was <5 in the Coastal Plains, well below our threshold set for use in validation.

**Fig. 4.**
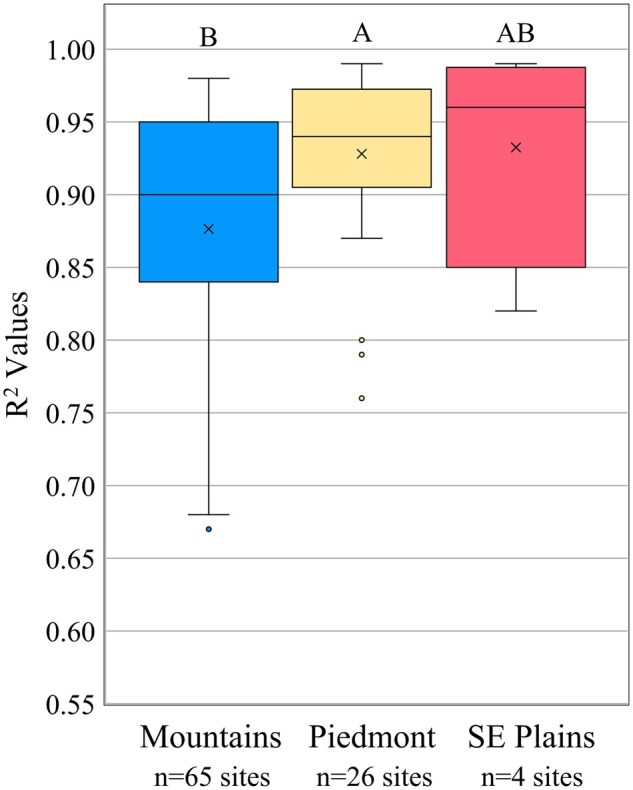
Boxplots of *R*^2^ values from proportion of F1 adult trap captures regressed against F1 adult model predictions in the Mountains, Piedmont, and Southeastern (SE) Plains using general linear models. Boxes represent the interquartile range, and inside the interquartile range, box mean is represented by X and the median by the horizontal line. Outside of the interquartile range box, vertical lines represent whiskers extending to the minimum and maximum values, excluding outliers. Dots represent outliers, that is, data points >1.5 times the length of the interquartile range box. General linear models were used to compare *R*^2^ among ecoregions, with ecoregions with different letters being significantly different.

**Fig. 5.**
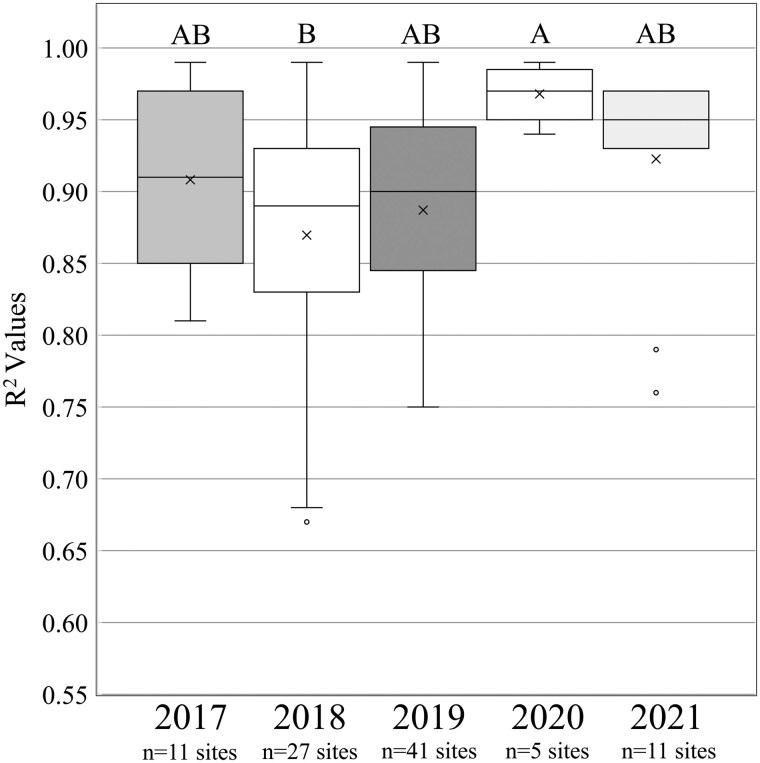
Boxplots of *R*^2^ values from proportion of F1 adult trap captures regressed against F1 adult model predictions by year from 2017 to 2021. Boxes represent the interquartile range, and inside the interquartile range, box mean is represented by X, and the median by the horizontal line. Outside of the interquartile range box, vertical lines represent whiskers extending to the minimum and maximum values, excluding outliers. Dots represent outliers, that is, data points >1.5 times the length of the interquartile range box. General linear models were used to compare *R*^2^ among years.

Regressions of cumulative proportion of F1 adult sticky trap captures over degree days versus model predictions were significant and had high *R*^2^ values for each site, validating model predictions for F1 adult eclosion. The GLM analysis of these *R*^2^ values as a function of region and year showed significant effects for region and year (Table 1). *R*^2^ values were higher in the Piedmont (*x̅* = 0.93 ± 0.012 SE) than the Mountains (*x̅* = 0.88 ± 0.009 SE), while the Southeastern Plains (*x̅* = 0.93 ± 0.039 SE) did not differ from the other 2 ecoregions (Fig. 4). *R*^2^ values in 2020 were significantly higher than in 2018 and did not differ significantly between these years and 2017, 2019, and 2021 (Fig. 5).

**Table 1.** GLM results for analyses for differences in *R*^2^ values resulting from observed proportional pheromone trap captures versus model-based predictions of F1 adult and nymphal populations among regions and years and F2 adult among years.

Analysis	Factor	*F*	*P*
F1 Adult *R* ^2^	**GLM model**	3.84_6,88_	**0.0019**
	**Region**	5.84_2,88_	**0.0041**
	**Year**	2.95_4,88_	**0.0245**
F2 Adult *R* ^2^	GLM model	1.23_3,12_	0.3402
F1 Nymphal *R* ^2^	**GLM model**	2.70_6,32_	**0.0310**
	**Region**	4.87_2,32_	**0.0142**
	Year	2.32_4,32_	0.0777

Significant factors are in bold.

#### F2 Adults

The Piedmont and the Southeastern Plains were the only regions with adults captured after 1356 DD and thus considered to be F2 adults. There were 30 sites by year with adequate F2 adult sticky trap captures to use for F2 adult model validation. Only 16 of the 30 site-year regressions of cumulative proportion F2 adult trap captures versus F2 adult model predictions were significant, and these only occurred in the Piedmont. The *R*^2^ values from these 16 models were low (*x̅* = 0.69 ± 0.021) compared to F1 adult model validation *R*^2^ values. The general linear model analyzing the 16 F2 adult *R*^2^ values for differences among years was not significant (Table 1).

### Oviposition Model Validation

**Fig. 6.**
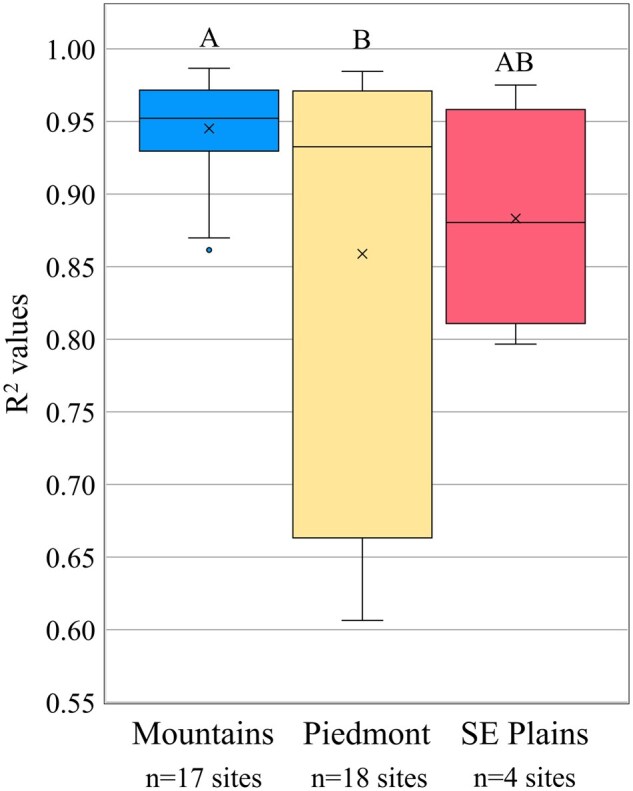
Boxplots of *R*^2^ values from proportion of F1 nymphal trap captures regressed against F1 nymphal oviposition model predictions in the Mountains, Piedmont, and Southeastern (SE) Plains ecoregions of NC. Boxes represent the interquartile range, and inside the interquartile range box mean is represented by X and the median by the horizontal line. Outside of the interquartile range box, vertical lines represent whiskers extending to the minimum and maximum values, excluding outliers. Dots represent outliers, that is, data points >1.5 times the length of the interquartile range box. General linear models were used to compare *R*^2^ values among regions, ecoregions with different letters being significantly different.

There were 17, 18, 4, and 0 sites in the Mountains, Piedmont, Southeastern Plains, and Coastal Plain, respectively, with adequate nymphal sticky trap captures (≥40 nymphs) to be used for F1 oviposition model validation. The regressions of cumulative proportion F1 nymphal trap capture over degree days against cumulative proportion nymphal model predictions were all significant, with high *R*^2^ values. The GLM analysis of sticky trap *R*^2^ values as a function of region and year showed that region was significant, but year was not (Table 1). The *R*^2^ values were higher in the Mountains (*x̅* = 0.95 ± 0.008) than the Piedmont (*x̅* = 0.86 ± 0.034) and did not differ significantly between the Southeastern Plains and the other 2 ecoregions (Fig. 6). Mean *R*^2^ values were all >0.85.

### Pyramid Versus Sticky Traps

**Fig. 7.**
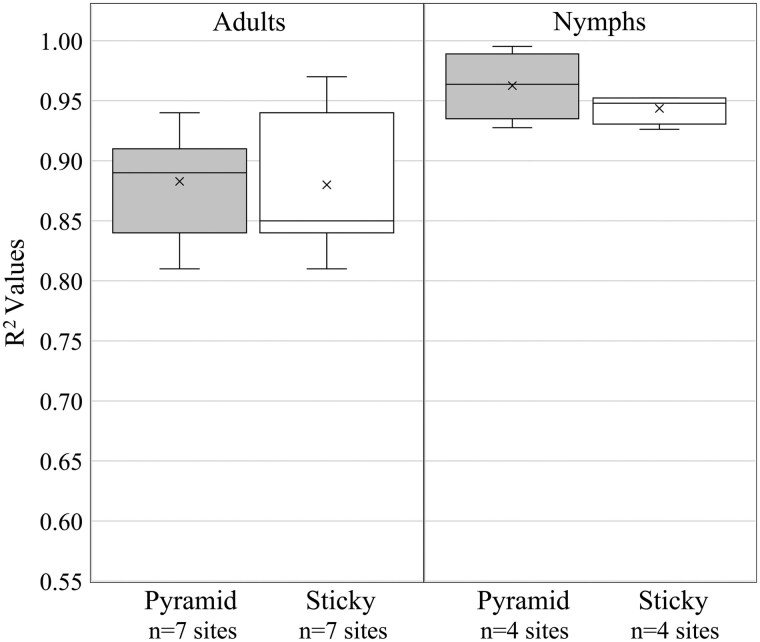
Boxplots of *R*^2^ values representing proportion capture of F1 adults and nymphs in different trap types regressed against F1 model proportion predictions. Boxes represent the interquartile range, and inside the interquartile range box mean is represented by X and median by the horizontal line. Outside of the interquartile range box, vertical lines represent whiskers extending to the minimum and maximum, excluding outliers. Dots represent outliers, that is, data points >1.5 times the length of the interquartile range box.

There were no significant differences when comparing *R*^2^ values of model predictions versus trap captures in 2017 when using pyramid and sticky traps for adults (*F*_1,12_ = 0.01, *P* = 0.919) or nymphs (*F*_1,6_ = 1.52, P = 0.264) (Fig. 7).

## Discussion

This study validated temperature-dependent models developed by Ogburn et al. (2023) for predicting adult emergence and oviposition of F1 BMSB by using extensive pheromone trap capture data from NC ecoregions. In addition to temperature, these ecoregions also differ in rainfall, elevation, and host plant availability. Nonetheless, there was a high level of agreement between temperature-based model predictions and pheromone trap captures; 86% and 60% of all adult regressions and 74% and 87% of all nymphal regressions had *R*^2^ values greater than 0.80 and 0.90, respectively. Trap data from the eastern Atlantic Coastal Plains ecoregion were too low for validation purposes, confirming that BMSB populations are not well established in this ecoregion (Bakken et al. 2015, Leskey et al. 2015, Venugopal et al. 2016, Ogburn et al. 2023). The genetic similarity among populations in eastern North America (Xu et al. 2014, Valentin et al. 2017) and their response to temperature and photoperiod (Nielsen et al. 2017, McDougall et al. 2021) suggest these models should be relevant throughout this region. Similarity of responses between eastern and western US populations is less clear (Nielsen et al. 2017, Mermer et al. 2023).

**Fig. 8.**
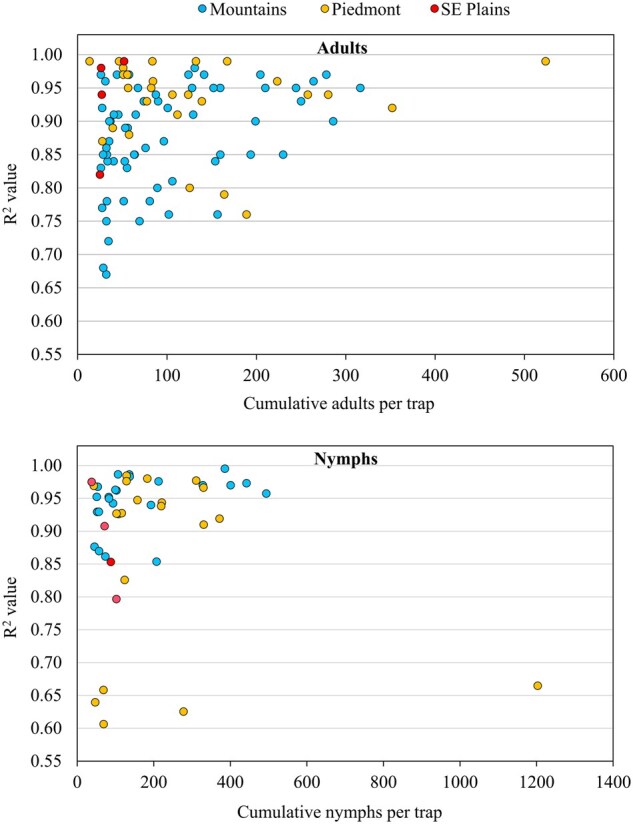
R^2^ values from regressions of *H. halys* adult and nymphal model predictions versus pheromone trap captures at different sites in the Mountains, Piedmont, and Southeastern (SE) Plains ecoregions of North Carolina. Correlation coefficient for adults was *r* = 0.251 (*P* = 0.012) and for nymphs *r* = 0.14 (*P* = 0.343).

There was variation among ecoregions in how well model predictions fit trap captures, with *R*^2^ values significantly higher for Piedmont versus Mountain adult regressions, and for Mountain versus Piedmont nymphal regressions. Greater variation in those sites with overall lower *R*^2^ values, but significant regressions, appeared to be associated with sites having low *H. halys* population densities (Fig. 8). Sites having low trap captures may have been isolated areas unsuitable for local establishment of *H. halys* (Hadden et al. 2022, Tamburini et al. 2023), possibly due to lack of suitable hosts, and captures may have been immigrants arriving independent of local phenological events. An outlier exception was the low *R*^2^ value for the highest nymphal population in the Piedmont. However, all regressions of cumulative trap capture versus model predictions were highly significant, and average *R*^2^ values were >0.85 across all ecoregions for F1 adult and nymphal models. This demonstrates the value of the models for predicting adult and nymphal emergence across all ecoregions, but precision may be reduced at sites with low population densities.

The difficulty in collecting sufficient BMSB eggs across diverse landscapes to directly validate the F1 oviposition model was overcome by using model predictions of oviposition in combination with stage-specific nymphal developmental and mortality rates from Nielsen et al. (2008) to predict nymphal populations over time and comparing these predictions with the cumulative number of nymphs caught in the traps. The high degree of correlation between predicted and observed nymphs in pheromone trap captures validated this approach. These results also suggest that mortality rates of immature field populations were consistent with the laboratory-observed rates reported by Nielsen et al. (2008) and that natural mortality in the field was inconsequential for modeling purposes. This is also consistent with low levels of biological control of BMSB eggs (the most vulnerable life-stage) often observed in field studies in its invaded range (Cornelius et al. 2016, Ogburn et al. 2016, Morrison et al. 2016, Dieckhoff et al. 2017, Pezzini et al. 2018, Costi et al. 2019, Ogburn et al. 2021). To our knowledge, this is the first report of a degree-day model constructed using data collected in natural settings to predict BMSB nymphal populations. However, due to generation overlap where 2 generations occur, its utility may be restricted to locations with only one generation per year (Nielsen et al. 2017).

The model predicting F2 adult eclosion did not fit adult trap capture data well. Only the Piedmont ecoregion had adequate adult trap captures after 1356 DD and could be categorized as F2 adults in this study. The F2 model was based on semi-field studies conducted in the Southeastern Coastal Plain (Goldsboro, North Carolina), where conditions were not favorable for BMSB establishment, greatly limiting the number of F2 adult samples on which the model was based and likely its robustness (Ogburn et al. 2023). Similar semi-field studies in the Piedmont region of NC, where BMSB is well established and 2 generations occur, perhaps could be used to develop a more robust F2 adult eclosion model. Regardless, developing validated models for predicting the F1 generation is the most useful tool for BMSB management in many eastern US crops.

Insect phenology models are valuable tools for studying the ecology and spread of invasive species and for informing pest management decisions. Indeed, phenology models are important components of invasive pest establishment forecast models (Bale et al. 2002, Zhu et al. 2012, Fraser et al. 2017, Kriticos et al. 2017), improving efficiency of surveillance, eradication and suppression programs (Barker et al. 2020, Crimmins et al. 2020, Barker and Coop 2024). They are also valuable for predicting voltinism and life-stage specificity across diverse climates (Nielsen et al. 2016) and forecasting effects of climate change on the range of distribution and biological control (Logan et al. 2006, Illán et al. 2022, Zhu et al. 2025). From a pest management perspective, temperature-driven insect phenology models have been widely used to optimize efficiency by timing management activities with peak susceptibility of the pest. For example, a degree-day phenology model to time insecticide applications with predicted egg hatch of the codling moth (*Cydia pomenella* L.) has been a key component of apple IPM programs for almost 50 yr (Croft et al. 1976, Riedl et al. 1976, Riedl and Croft 1978). While this original model has been updated to account for variation in the response of differing geographic populations (Jones et al. 2008, Chappell et al. 2015), it remains the foundation for timing pesticide applications against codling moth. Historical and real-time weather data are easily accessible through web sources for use as a model input to rapidly forecast pest activity and have facilitated the use of phenology models for multiple species by pest management specialists and growers (Jones et al. 2010, Barker et al. 2020, Crimmins et al. 2020, Barker and Coop 2024). Finally, phenology models are tools that can also be used to assess the efficacy of management programs (Rincon et al. 2024).

BMSB F1 adults are the most damaging life stage on numerous crops in much of the eastern United States, especially tree fruits (Nielsen and Hamilton 2009, Joseph et al. 2015, Zobel et al. 2016). The validated F1 adult eclosion model enables insecticide applications for BMSB to be timed to coincide with the beginning of adult emergence (5% to 10% eclosion) to prevent damage to apples, which occurs soon after adult emergence begins in late summer. The precise timing could be further refined with the use of action thresholds (Short et al. 2017). The validated F1 oviposition model also provides a tool for timing releases of the BMSB egg parasitoid *Trissolcus japonicus* (Ashmead). Adventive populations of this Asian parasitoid were first detected in the mid-Atlantic states of the eastern United States in 2014 (Talamas et al. 2015). Releases of *T. japonicus* have shown promise for post-release establishment of *T. japonicus* in BMSB’s invaded range (Lowenstein et al. 2019, Falagiarda et al. 2023). Timing area-specific releases of *T. japonicus* to coincide with the predicted timing of first or peak BMSB oviposition would synchronize the presence of female parasitoids with the presence of host eggs in the environment.

These BMSB phenology models are expected to support improved management planning for this important agricultural pest. Temporal predictions can reduce dependence on resource-intensive sampling programs, improve the efficiency of insecticide use to suppress damage on susceptible crops, and forecast changes in populations associated with chemical and biological control programs.
